# Efficacy of perioperative duloxetine as a part of multimodal analgesia in laparoscopic colorectal cancer surgeries

**DOI:** 10.1186/s12871-023-02119-8

**Published:** 2023-05-16

**Authors:** Diab Fuad Hetta, Montaser Abdelfatah Mohamed, Hany Ahmed Elmorabaa, Mirna Ismail Ahmed, Nourhan Alaa Elgalaly, Shereen Mamdouh Kamal

**Affiliations:** 1 Anesthesia and intensive care and pain management department, South Egypt Cancer Institiue, Assiut University, Assiut, Egypt; 2 Anaesthesia, Intensive care and pain management department, Faculty of Medicine, Assiut University, Assiut, Egypt

**Keywords:** Perioperative, Duloxetine, Multimodal analgesia, Laparoscopic, Colorectal cancer surgery

## Abstract

**Background:**

Although laparoscopic surgery provides earlier recovery, less morbidity and hospital stay, however, severe pain is still a problem after it. Duloxetine has been recently used in postoperative pain management. We tested perioperative duloxetine to evaluate its effect on patients undergoing laparoscopic colorectal cancer surgery.

**Methods:**

Sixty patients were enrolled in this study divided into two equal groups; duloxetine group each patient received an oral duloxetine capsule (60 mg) 1st dose at night before surgery, the 2nd dose 1 h preoperative, and the 3rd dose 24 h postoperative. Placebo group received placebo capsules at the same times. The cumulative morphine consumption in 48 h, postoperative VAS score, quality of recovery (QoR-40 score), sedation, and adverse effects were evaluated.

**Results:**

Duloxetine group had lower VAS scores compared to placebo group, (3 ± 0.69) VS. (4.17 ± 0.83), (2.5 ± 0.6) VS. (4.3 ± 0.9), (2.2 ± 0.7) VS. (3.9 ± 0.6), (1.6 ± 0.7) VS. (3.6 ± 0.8), (1.1 ± 0.8) VS. (3.7 ± 0.7), (0.7 ± 0.7) VS. (3.5 ± 0.8), (0.6 ± 0.7) VS. (3.5 ± 0.8) respectively, P ˂0.01. The cumulative morphine consumption was significantly reduced in the Duloxetine group compared to the placebo group (4.6 ± 2.9 vs. 11.3 ± 1.7 mg), P < 0.01. The total QoR-40 score for duloxetine group was (180.8 ± 4.5) vs. (156 ± 5.9) in placebo group (*P* < 0.01). Patients in Duloxetine group were more sedated in all the 48 h postoperatively in comparison to placebo group.

**Conclusions:**

Perioperative duloxetine had reduced postoperative pain, decreased opioid consumption, and improved the quality of recovery in patients undergoing laparoscopic colorectal surgery.

## Introduction

GLOBOCAN 2018 reported that colorectal cancer (CRC) is the third most deadly and fourth-most common cancer worldwide [1]. Surgery is always the primary line of treatment in early-diagnosed cases but it is no longer effective in advanced cases where cancer has metastasized, as is the case in about 25% of diagnoses[2]. Abdominal surgery is usually accompanied by severe postoperative pain. The greater propensity for pain and opioid-related side effects are likely contributing factors for poor postsurgical recovery, and it often results in significant pain and slow recovery [3]. The laparoscopic approach is increasingly utilized, as it is associated with decreased postoperative pain and morbidity, as well as earlier recovery and a shorter hospital stay when compared with open surgery. Even so, pain may still be relatively severe, especially in the early postoperative period [4].

Multi-modal analgesia is advocated for perioperative pain management, to reduce opioid use and its associated adverse effects [5]. Multi-modal analgesia can be achieved by combining different analgesics and different routes of administration to achieve better analgesia synergistically compared with conventional analgesia [6]. Therefore, lower doses of each drug can be provided with fewer overall side effects from individual compounds [7]. Serotonin and norepinephrine are concerned with the modulation of endogenous analgesic mechanisms through descending inhibitory pain pathways in the brain and spinal cord. An increase in serotonin and norepinephrine may increase the inhibition of nociceptive input and improve pain relief [8].

Duloxetine is a serotonin-norepinephrine reuptake inhibitor (SNRI) commonly prescribed for the treatment of major depression and anxiety; it has also been used in the treatment of chronic pain conditions [9]. Duloxetine has been recently introduced in the management of severe pain after major abdominal cancer surgery [10]. The beneficial characteristics of duloxetine on pain and emotions encouraged the researchers to use it in the perioperative period [11, 12].

We hypothesized that the incorporation of perioperative oral duloxetine doses would produce a reduction in postoperative pain intensity, opioid consumption, and quality of recovery for patients subjected to colorectal surgery.

## Patients and methods

### Ethics approval and consent for participation

This study is a prospective, randomized, double-blinded, placebo-controlled trial. It was done after obtaining approval from both the local ethics committee and the Institutional Review Board (IRB) from the South Egypt Cancer Institute, Assuit University, Assuit, Egypt and strictly followed the regulations and amendments of Helsinki Declaration. Informed written consent was obtained from all patients. The study was recorded at Clinical trials, identifier: NCT04294953 at 4/3/2020, and conducted according to the Consolidated Standards of Reporting Trials (CONSORT) statement.

### Participants

The inclusion criteria were: adult patients with ASA I-III, aged 18–65 years and, scheduled for laparoscopic colorectal surgery for cancer colon. The exclusion criteria were: Patients that have an allergy to the study drug, abnormal liver or renal function tests, a chronic opioid user (> 3 months), being on gabapentin or pregabalin (> 3 months), antidepressant drugs, patients with psychiatric disorders, pregnant females, unable to express their pain or patient refusal.

### Outcome

The primary aim of our study is to evaluate the efficacy of perioperative duloxetine on postoperative morphine consumption after colorectal surgery. The secondary aims include its effect on VAS pain scores and postoperative quality of recovery.

### Randomization and blinding

Sixty patients were randomized by the use of a computer-generated table of random numbers in a 1:1 ratio into 2 groups and concealed in an opaque and sealed envelope.

#### Duloxetine group

Each patient received an oral duloxetine capsule of 60 mg 12 h before surgery, the 2nd dose of 60 mg 1 h preoperative and, the 3rd dose of 60 mg 24 h postoperative.

#### Placebo group

Each patient received a placebo capsule at the same scheduled times. The placebo capsule was prepared by the hospital pharmacy to ensure that active duloxetine and placebo capsules were identical.

The patient’s follow-up was conducted by an anaesthesia resident blinded to the study. The attending anaesthesiologist, surgeon and data-collecting person were unaware of the patient assignment.

All patients were instructed on how to evaluate their pain using VAS (Visual Analog Scale) [13] scoring from 0 to 10 where 0 = no pain and 10 = the worst pain imaginable and how to use PCA (Patient Controlled Analgesia) device. The quality of Recovery questionnaire [14] was explained to each patient to facilitate evaluating their physical comfort, emotional state, physical independence and pain. The questionnaire measures five components of patient recovery: physical comfort (12 questions), physical independence (5 questions), emotional state (9 questions), psychological support (7 questions), and, pain (7 questions). The sum of each component generates an aggregate score.

### Procedures

The anaesthetic protocol was standardized for all patients. Basic monitoring (pulse oximeter, electrocardiogram, non-invasive arterial blood pressure, and end-tidal Co2) was attached. IV access was inserted and 500 ml of normal saline was given preoperative. Anesthesia was induced with fentanyl (Fentanyl Hamein, Sunny Pharmaceutical, Germany) 2 µg/kg, propofol (Propofol 1%, Fresenius Kabi, Deutschland) 1–2 mg/kg, lidocaine 1.5 mg/kg and rocuronium (Esmerone, Organon, The Netherlands) 0.5 mg/kg was administered to facilitate endotracheal intubation. Anesthesia and muscle relaxant were maintained by isoflurane 1 to 1.5 MAC (minimum alveolar concentration) in a 50% oxygen/air mixture and rocuronium 0.15 mg/kg respectively. Additional intraoperative analgesia consisting of IV boluses of 25 µg fentanyl was given as determined by fluctuation in vital signs (increasesd in heart rate > 20% above the basal value). At the end of the surgery, a dose of paracetamol 1 gm/100 ml IV was given to all patients before extubation. Intravenous neostigmine (0.05 mg/kg) and atropine (0.01 mg/kg) were administered to reverse muscle relaxants.

After extubation, patients were transferred to the post-anesthesia care unit (PACU), where a standard analgesic regimen of paracetamol 1 g was given intravenously every 8 h. to all patients. In addition, an intravenous PCA morphine bolus of 3 mg without background infusion and a lockout period of 15 min were given upon patient’s request during the follow up period.

### Outcome assessments and data collection

In the PACU, the following parameters were assessed: (1) the cumulative morphine consumption in the first 48 h (as a primary outcome). (2) Pain intensity was evaluated by VAS at time intervals 0–2 h, 2–4 h, 4–8 h, 8–12 h, 12–24 h, 24–36 h, and 36–48 h. (3) Also, the quality of recovery − 40 scoring system (QoR-40) was recorded by the anesthesia resident 48 h after the surgery. (4) Postoperative sedation was assessed at 0–2, 2–4, 4–6, 6–12, 12–24, and 24–48, using a Modified Observer’s Assessment of Alertness */*Sedation Scale (MOSS/A) [15] where 6 *=* agitated, 5 = response readily to name spoken in normal tone (alert), 4 = lethargic response to name spoken in a normal tone, 3 = responds only after a name is called loudly and/or repeatedly, 2 = response only after mild prodding or shaking 1 = doesn’t respond to mild prodding or shaking, 0 = doesn’t respond to deep stimulus. (5) Postoperative adverse effects such as headache, nausea, vomiting, hypotension, bradycardia, respiratory depression, itching, and sedation were reported.

### Statistical analysis

#### Sample size calculation

The sample size was calculated using the G* power program. Based on a previous study [16] that detected a large effect size in the first postoperative 24 h morphine consumption between Duloxetine and placebo group, we used a priori Cohen’s d of 0.8, an alpha error of 0.05 and a study power of 90% to obtain a minimum sample size of 56 subjects, to allow for dropouts we included 60 patients, 30 in each group.

#### Statistical analysis

IBM-SPSS 24.0 (IBM-SPSS Inc., Chicago, IL, USA) was used for data verification, coding by the researcher and analysis. The normality of data distribution among the studied groups was tested with the Shapiro-Wilk and Kolmogorov-Smirnov tests. Normally distributed continuous data were reported as means ± (SD) and were compared with 2-sided independent t-tests for equal variances. Non- Normally distributed interval data and ordinal data were reported as medians (interquartile range [IQR]) and were compared using the T-test. Categorical variables are presented as counts (percentages) and were evaluated with the Fisher exact test. Nonparametric correlations using the Spearman rho correlation coefficient between the global QoR-40 and “both opioid consumption and the pain subcomponent of the QoR-40” were performed. The level of rejection of the null hypothesis, using the T-test was set at P value < 0.05 for the postoperative cumulative 48 h morphine consumption.

## Results

### Patients characteristics

**Fig. 1:**
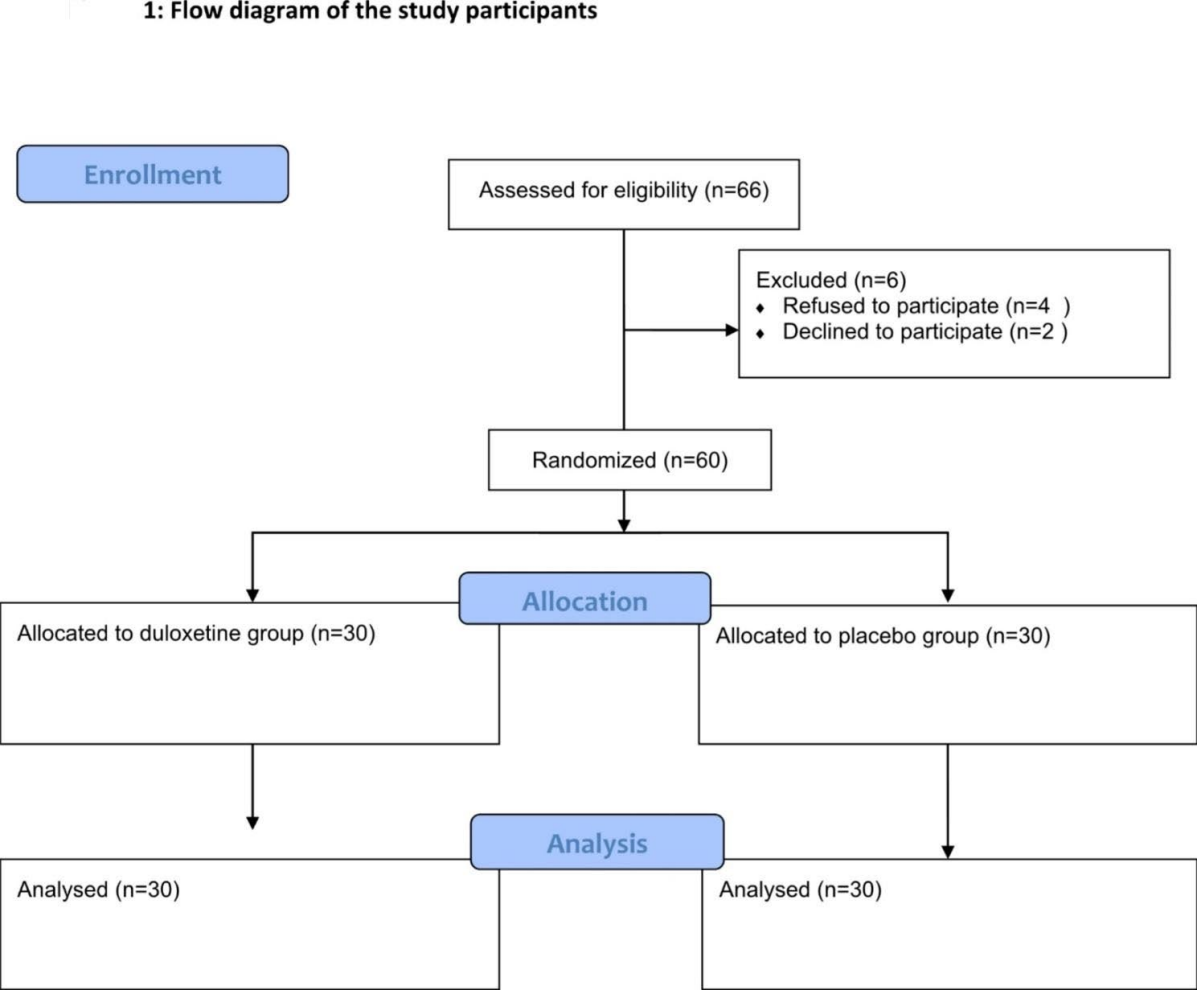
Flow diagram of the study participants

Sixty-six patients were assessed for eligibility to participate in the study. Four patients were inoperable and two patients refused to participate. A final sixty patients were scheduled for laparoscopic colorectal surgery for cancer colon completed this study and equally distributed in two groups (n = 30 patient per group) as shown in flow diagram (Fig. 1). The two groups were similar regarding baseline demographic data and patient characteristics (Table 1).

**Table 1:** Demographic data and patient’s characteristics

	Duloxetine Group (n = 30)	Placebo Group (n = 30)	P value
**Age/years**	45.83 ± 10.4	43.83 ± 6.4	0.375*
**Sex**			
**Female**	13 (43.3%)	10 (33.3%)	0.426**
**Male**	17 (56.7%)	20 (66.7%)	
**Weight/kg**	84.17 ± 12.5	80.67 ± 10.8	0.250*
**Height/kg**	163.37 ± 5.9	165.43 ± 4.4	0.131*
**BMI**	31.23 ± 3.8	29.72 ± 3.2	0.103*
**ASA**			
**I**	10 (33.3%)	15 (50%)	0.250*
**II**	15 (50%)	9 (30%)	
**III**	5 (16.7%)	6 (20%)	
**Operative duration (h)**	4.2 ± 0.95	4.3 ± 0.95	0.847
**Intravenous fluid (L)**	2.9 ± 0.60	2.9 ± 0.56	0.834

Data were represented as mean, standard deviation, number and percentage

*Independent t-test was used to compare the means among groups

### Study endpoints

#### Primary outcome

The duloxetine group had significantly lower pain scores represented by VAS score in the first 48 postoperative hours than the placebo group at all-time intervals: 0–2, 2–4, 4–8, 8–12, 12–24, 24–36 and,36–48, Mean and standard deviation (mean ± SD) (3 ± 0.69) vs. (4.17 ± 0.83), (2.5 ± 0.6) vs. (4.3 ± 0.9), (2.2 ± 0.7) vs. (3.9 ± 0.6), (1.6 ± 0.7) vs. (3.6 ± 0.8), (1.1 ± 0.8) vs.(3.7 ± 0.7), (0.7 ± 0.7) vs. (3.5 ± 0.8), (0.6 ± 0.7) vs. (3.5 ± 0.8) respectively, P value ˂0.01 (Table 2).

**Table 2:** Post-operative VAS score in 48 h, intraoperative fentanyl consumption, and post-operative total morphine consumption in 48 h

	Duloxetine Group (n = 30) (mean, SD)	Placebo Group (n = 30) (mean, SD)	Mean Difference (CI)	P value
**VAS 0–2 h**	3 ± 0.69	4.17 ± 0.83	-1.16 (-1.56, -0.77)	0.01˂
**VAS 2–4 h**	2.57 ± 0.67	4.30 ± 0.91	-1.73 (-2.15, -1.31)	0.01˂
**VAS 4–8 h**	2.23 ± 0.77	3.97 ± 0.76	-1.73 ( -2.13, -1.33)	˂0.01
**VAS 8–12 h**	1.63 ± 0.71	3.67 ± 0.84	-2.03 ( -2.43, -1.62)	˂0.01
**VAS 12–24 h**	1.17 ± 0.87	3.70 ± 0.75	-2.53 (-2.95, -2.11)	0.01˂
**VAS 24–36 h**	0.77 ± 0.77	3.53 ± 0.81	-2.76 (-3.17, -2.35)	˂0.01
**VAS 36–48 h**	0.60 ± 0.77	3.50 ± 0.86	-2.90 (-3.32, -2.47)	˂0.01
**Intraoperative fentanyl consumption**	25.33 ± 26.20	74.16 ± 27.45	-50.8(-64.7, -36.9)	< 0.01
**Total postoperative morphine consumption**	4.6 ± 2.9	11.3 ± 1.7	-6.7 (-7.9, -5.4)	< 0.01

Data were represented as mean, standard deviation, mean difference and 95% confidence interval of the difference

#### Secondary outcome

Total intraoperative fentanyl consumption was decreased in the duloxetine group compared to the placebo group, mean and standard deviation (mean ± SD) (25.33 ± 26.20) vs. (74.16 ± 27.45), P value ˂0.01 (Table 2).

The cumulative 48 h morphine consumption was significantly reduced in the Duloxetine group compared to the placebo group, (mean ± SD) (4.6 ± 2.9 mg vs. 11.3 ± 1.7 mg), P < 0.01 (Table 2).

Subjects in the duloxetine group had better postoperative quality of recovery than the placebo group. The mean and standard deviation (mean ± SD) of total QoR-40 score for the duloxetine group was (180.8 ± 4.5) compared to (156 ± 5.9) in the placebo group (*P* < 0.01) at 48 h postoperatively, moreover the individual subcomponents in the QoR-40 showed that the duloxetine group had better results than the placebo group in the psychological support, emotional status, physical comfort, physical independence and, pain (32.2 ± 1.7) vs. (29.2 ± 1.9), (41.9 ± 1.8) vs. (35.4 ± 3.4), (52.3 ± 1.7) vs. (47 ± 3.3), (22.1 ± 1.6) vs. (18.9 ± 1.3), (32 ± 1.6) vs. (25.3 ± 2.3) respectively, P value ˂0.01 (Table 3).

**Table 3:** Quality of recovery (QOR) questionnaire

	Duloxetine Group (n = 30) (Mean, SD)	Placebo Group (n = 30) (Mean, SD)	Mean Difference (CI)	P value
**Psychological support**	32.2 ± 1.7	29.2 ± 1.9	3 (2.1, 4)	0.01˂
**Emotional state**	41.9 ± 1.8	35.4 ± 3.4	6.5 (5, 7.9)	0.01˂
**Physical comfort**	52.3 ± 1.7	47 ± 3.3	5.3 (3.9, 6.7)	0.01˂
**Physical Independence**	22.1 ± 1.6	18.9 ± 1.3	3.2 (2.4, 4)	0.01˂
**Pain**	32 ± 1.6	25.3 ± 2.3	6.7 (5.6, 7.7)	0.01˂
**Total**	180.8 ± 4.5	156 ± 5.9	24.7 (22, 27.4)	0.01˂

Data were represented as mean, standard deviation, mean difference and 95% confidence interval of the difference

Patients in the Duloxetine group appeared to be more sedated in all the 48 h postoperatively in comparison to the placebo group. Median (IQR) of Modified Observer’s Assessment of Alertness */*Sedation Scale (MOSS/A) at 0–2, 2–4, 4–6, 6–12, 12–24 and, 24–48 was 3 (3–4) vs. 4 (4–5), 3 (3–4) vs. 4 (4–5), 3 (2–4) vs. 4 (4–5), 3 (2–4) vs. 4 (4–5), 3 (3–4) vs. 4 (4–5) and, 3 (3–4) vs. 4 (4–5), P value ˂0.01 (Table 4).

**Table 4:** Post-operative sedation score by Modified Observer’s Assessment of Alertness */*Sedation Scale (MOSS/A)

	Duloxetine Group (n = 30) Median (IQR)	Placebo Group (n = 30) Median (IQR)	P value
**0–2 h.**	3 (3–4)	4 (4–5)	˂0.01
**2–4 h.**	3 (3–4)	4 (4–5)	˂0.01
**4–6 h.**	3 (2–4)	4 (4–5)	˂0.01
**6–12 h.**	3 (2–4)	4 (4–5)	˂0.01
**12-24 h.**	3 (3–4)	4 (4–5)	˂0.01
**24-48 h.**	3 (3–4)	4 (4–5)	˂0.01

Data were represented as median (IQR).

Concerning the postoperative side effects during the first 48 h, where number and percentage of nausea, vomiting, bradycardia and headache were more in the duloxetine group than the placebo group, 6 (20%) vs. 2 (6.6%), 5 (16.6%) vs. 2 (6.6%), 1 (3.3%) vs. 0 and, 3 (10%) vs. 1 (3.3%) respectively; howerver they did not reach the statistical significance level (Table 5).

**Table 5:** Post-operative side effects

	Duloxetine Group (n = 30)	Placebo Group (n = 30)	P value
**Nausea**	6 (20%)	2 (6.6%)	0.05
**Vomiting**	5 (16.6%)	2 (6.6%)	0.11
**Itching**	2 (6.6%)	2 (6.6%)	0.50
**Bradycardia**	1 (3.3%)	0	0.17
**Headache**	3 (10%)	1 (3.3%)	0.13

Data were represented as number and percentage (%)

## Discussion

The present trial had shown that patients subjected to laparoscopic colorectal surgery who administered three perioperative duloxetine, 60 mg 12 h preoperative, a second dose one hour preoperative, and a third dose 24 h postoperative, had a significant lower postoperative morphine consumption, intraoperative fentanyl consumption, a significant decreased postoperative pain and better quality of recovery for 48 h postoperatively. The adverse effects including nausea, vomiting, bradycardia, and headache were higher in the duloxetine group compared with the placebo group; however they did not reach the statistical significant level.

The unique criteria of our study that administered three perioperative doses of duloxetine instead of single or twice doses as in previous studies, more over we assess the perioperative duloxetine on the QoR. Furthermore, the nature of pain in laparoscopic surgery is different from open surgery.

Unlike postoperative pain after open surgery, which is most of somatic origin, postoperative pain after laparoscopic surgery consists of both somatic and visceral elements [17]. The somatic pain following laparoscopic procedures is a sharp pain that is usually localized in the abdomen [18]. The mechanisms behind this pain, as highlighted by Gough and his colleagues [19], are the perforation of the abdominal wall and the insertion of trocars, sutures, and tacks. Visceral pain, which has been overlooked in Gough’s study [19], is referral in nature and causes moderate to severe dull pain in the shoulder, scapula, and abdomen. Visceral pain after laparoscopic surgery can be activated by the traction of the peritoneum or by diaphragm irritation after surgical manipulation, intraoperative gas insufflation, and postoperative gas retention [18–20].

The improved postoperative analgesia, reduction of opioid consumption and pain intensity reported in our study is in concordance with Castro-Alves and his collegues [21], they detected a reduction in pain scores, opioid consumption, and improvement in the quality of recovery after administration of duloxetine (60 mg orally 2 h before and 24 h after hysterectomy) in comparison with placebo pill; however, these effects were limited to the first 24 h postoperatively.

Moreover, in our previous [10] trials on the role of perioperative duloxetine in major abdominal cancer surgery, we reported a reduction in the cumulative 48 h morphine consumption in the duloxetine group compared to the placebo group, (5.2 mg ± 3.2 Vs 12.9 mg ± 3.4), mean difference (95% CI) 7.6 mg (5.9–9.3) P < 0.001. Also, in this trial subjects in the duloxetine group had a better postoperative quality of recovery than the placebo group. The median (IQR) of the global QoR-40 score for the duloxetine group was 185 (180–191) compared to170 (163–175) in the placebo group (*P* < 0.001). Furthermore, Ho and his co-workers [22] gave two doses of duloxetine 60 mg on successive days after knee replacement surgery and found a significant reduction in cumulative morphine consumption 48 h postoperative, in the first 24 h (12.9 ± 10.4 mg vs. 19.8 ± 13.7 mg) and 48 h (19.5 ± 14.5 mg vs. 30.3 ± 18.1 mg) postoperative. This agrees with our study that revealed a significant reduction in morphine consumption in the duloxetine group (4.6 ± 2.9 mg VS 11.3 ± 1.7 mg) in the placebo group 48 h postoperative. However, the postoperative VAS score was similar in both groups in their study.

It is reasonable that if an analgesic could also provide emotional stability, it would be a better adjunct for postoperative recovery. One of the most important findings in our study was the better postoperative quality of recovery, as represented by the total score of 180.8 ± 4.5 in patients who received duloxetine compared with those who received a placebo capsule 156 ± 5.9. It specifically improved the five domains: physical comfort, independence, emotional, psychological, and pain in the quality of recovery score. This improvement in QOR is not only due to less opioid consumption and lower VAS scores but also due to a favorable effect on emotional and psychological status which are of utmost importance in cancer patients undergoing surgery.

Nasr’s ***s***tudy; administered Duloxetine 60 mg 2 days before surgery till 2 weeks postoperatively in patients undergoing mastectomy and found that it not only reduced pain postoperatively but also improved chronic pain at 3 months and 6 months postoperatively [23].

Also, in accordance with our study, a meta-analysis conducted by Zorrilla and partners [24] approved the effectiveness of the perioperative use of Duloxetine for the treatment of acute postoperative pain. Altiparmak and co-workers [25] compared pregabalin and duloxetine in their role as adjuvants in a multimodal analgesia regime and postoperative effects on cognitive function after spinal surgery. They found that the analgesic efficacy of duloxetine and pregabalin were similar and significantly greater than a placebo.

In contrast to our study, Erdmann and his collegues [26] studied the effects of a short-term perioperative duloxetine treatment on 60 patients undergo open colectomy surgery and they concluded that duloxetine did not reduce total opioid consumption or pain intensity during the initial 48 h following major colon surgery. They found reduction in opioid consumption and VAS score but not reach the statistical significance level as they administered only two doses for operation (open colectomy) with known sever postoperative pain.

Dose selection in this trial was inspired by our previous study [10], where the patients received a single dose of duloxetine, 60 mg preoperative. We assumed that increasing the duloxetine dose to three perioperative doses will give better results represented in morphine consumption, pain intensity, and quality of recovery.

It has been proposed that duloxetine exerts its analgesic action through three different processes and locations. The dorsal horn of the spinal cord dopamine, NE, and serotonin levels are raised as a result of its action. These monoamines stimulate the spinal cord’s 5-HT_2A_ and aplha2-norepinephrine receptors, which strengthen the inhibitory descending pain pathways. Prefrontal brain activity, which results in cognitive control of pain, is another central mechanism [27, 28].

### Study limitations

Follow up period was only 48 h postoperatively; we could not report the effect of duloxetine on chronic pain management. We did not report the effect of duloxetine on the hospital stay period or the intestinal recovery. In further studies, the third group with two doses of Duloxetine group could be added to detect the effect of the dose on the side effects.

## Conclusion

The perioperative doses of duloxetine 60 mg, the first 12 h preoperatively, a second dose one hour preoperatively, and a third dose 24 h postoperatively had reduced postoperative pain, decreased opioid consumption, and improved the quality of recovery for 48 h postoperatively in patients subjected to laparoscopic colorectal surgery.

## Data Availability

The database is closed and there is no public access. However, permission to access and use the database can be obtained if necessary by request to the corresponding author.

## Abbreviations

ASA: American Socity of Anesthesiology physical status classification
CI: Confident interval
CRC: Colorectal cancer
IRB: Institutional Review Board.
IOR: Interquartil range
MOSS/A: Modified Observer’s Assessment of Altreness/ Sedation Scale
NSAIDS: Non steroidal antiinflammatory drugs
SNRI: Seretonin-norepinephrine reuptake inhibitor.
PCA: Patient controlled analgesia
PACU: Postanesthesia care unit
QoR-40: The Quality of Recovery − 40 scoring system
VAS: Visual Analog Scale
5HT_2A_: 5 Hydroxy-tryptamine_2A_
